# Design of chemobrionic and biochemobrionic scaffolds for bone tissue engineering

**DOI:** 10.1038/s41598-024-63171-z

**Published:** 2024-06-14

**Authors:** Bahar Aslanbay Guler, Zehra Gül Morçimen, Şeyma Taşdemir, Zeliha Demirel, Ezgi Turunç, Aylin Şendemir, Esra Imamoglu

**Affiliations:** 1 Bioengineering Department, Faculty of Engineering, Ege University, Izmir, Turkey; 2 Ioengineering Department, Faculty of Engineering, Manisa Celal Bayar University, Manisa, Turkey; 3 Department of Biochemistry, Faculty of Pharmacy, İzmir Katip Çelebi University, İzmir, Turkey

**Keywords:** Biological techniques, Cell biology, Stem cells, Medical research, Materials science

## Abstract

Chemobrionic systems have attracted great attention in material science for development of novel biomimetic materials. This study aims to design a new bioactive material by integrating biosilica into chemobrionic structure, which will be called biochemobrionic, and to comparatively investigate the use of both chemobrionic and biochemobrionic materials as bone scaffolds. Biosilica, isolated from *Amphora sp*. diatom, was integrated into chemobrionic structure, and a comprehensive set of analysis was conducted to evaluate their morphological, chemical, mechanical, thermal, and biodegradation properties. Then, the effects of both scaffolds on cell biocompatibility and osteogenic differentiation capacity were assessed. Cells attached to the scaffolds, spread out, and covered the entire surface, indicating the absence of cytotoxicity. Biochemobrionic scaffold exhibited a higher level of mineralization and bone formation than the chemobrionic structure due to the osteogenic activity of biosilica. These results present a comprehensive and pioneering understanding of the potential of (bio)chemobrionics for bone regeneration.

## Introduction

The critical bone defects resulting from trauma, infection, or diseases are of significant worry due to their negative effects on patient’s life quality. Bone tissue engineering aims to repair the damaged tissues by creating suitable conditions to guide the natural bone regeneration process. The main step of this period is the development of three-dimensional (3D) scaffolds, which mimic the extracellular matrix (ECM) of bone, provide mechanical support and eventually form new functional tissue. The ideal bone scaffolds should have appropriate properties in terms of surface texture, porosity, mechanical strength, and biocompatibility, should promote cell growth, and degrade following the new bone formation without any adverse effects ^1,2^. Besides these essential characteristics, improving the chemical composition of scaffolds by incorporating components that enhance the osteoinduction and/or osteoconduction capacities of the material is a promising strategy to achieve greater scaffold bioactivity. Silica (Si), which is a minor constituent of the bone, has been defined as an effective supplement that supports osteogenesis and improves the osteoinductive properties of scaffold ^3,4^. It has been reported that Si incorporated scaffolds enhance biomineralization and bone tissue regeneration by means of increasing the surface deposition of biologically active layers and upregulating the gene responsible for osteogenesis ^5^. Recently, one of the most outstanding examples of natural silica sources has been diatoms, which are major contributors to the global silica cycle. The amorphous silica shell of diatoms, which are called frustules, have some unique properties, including high mechanical stability, well-arranged porous structure, large surface area, high absorption capacity, and low thermal conductivity. These specific characteristics have pointed out the use of diatom silica in engineering and medical fields, especially for the bone tissue engineering applications ^6,7^. When comparing diatom biosilica (BS) to synthetic silica for bone applications, several advantages become apparent. Diatom BS exhibits a rough surface and porous structure, which can enhance cell adhesion and proliferation. Moreover, it possesses a faster resorption rate than synthetic silica. Additionally, diatom BS offers a more sustainable source of silicon-based materials, as its synthesis does not require the use of harsh chemicals, conditions, or energy-intensive processes ^8^.

There have been many types of bone scaffolds (e.g., ceramic, metal, collagen, chitin, polylactic-glycolic acid, polycaprolactone, etc.) produced by different methods such as, freeze-drying, particulate leaching, solvent casting, gas foaming, sol–gel, electrospinning, and 3D-bioprinting ^2,9^. Among the various production techniques, self-organization is another promising strategy to design and construct functional scaffolds because of its simplicity, versatility, and cost-effectiveness. It describes the spontaneous organization of small molecules or ions into well-defined structures on the basis of various physical principles including chemical stimuli, gravity, temperature, magnetic field, buoyancy, and osmotic forces ^10,11^. This process is a highly common phenomenon in nature, covering a number of hierarchically self-organized systems such as honeycomb, sea shells, Liesegang patterns, the Belousov–Zhabotinsky reaction, and chemobrionics ^12,13^.

Chemobrionics is a scientific field that covers different self-organizing complex systems and related phenomena. Chemical gardens are one of the most common and fascinating examples of chemobrionics that are grown by the precipitation reaction of ions from metal salts and anionic solutions. Seeding a salt crystal or injecting its solution into an anionic solution produce tubular mineral structures in different shapes at micro- and macro levels ^14,15^. Investigations of the physical, chemical, and morphological properties of these structures have implicated the potential application areas of chemobrionics, including geology ^16^, electrochemistry ^17^, organic chemical gardens ^18^, microfluidic systems ^19^, and material science ^20^. From a medical perspective, chemobrionics has attracted substantial attention due to their ability to mimic bone tissue in terms of morphology (structural similarity to osteons, Haversian canals, etc.) and chemical composition (tubes with calcium, silicate, phosphate). Depending on the used chemicals, chemobrionics tend to show similar compositions to ceramic, hydroxyapatite, and glass biomaterials. The potential of chemobrionics to support cellular attachment and promote cell growth was reported for different cell strains such as choanoflagellate *Salpingoeca rosetta*, dinoflagellate *Pyrocystis lunula*, mammalian fibroblasts H9C2 ^21^, but a highly limited number of studies investigated the use of these structures as bone scaffolds ^22,23^.

Two main aims of this study were: (i) to design a bioactive bone scaffold, which will be called biochemobrionic (BCB), by integrating BS into the chemobrionic (CB) structure, (ii) to investigate the use of both CB and BCB materials as bone scaffolds. Based on previous reports, it was hypothesized that CB material could serve as a suitable scaffold for cell proliferation and bone tissue regeneration because of its biomimicry to natural bone. Additionally, the integration of BS into the CB structure was expected to enhance osteogenesis due to the advantages of BS over synthetic silica, providing novel insights into the potential of the BCB material for bone tissue engineering. To test these hypotheses, the BS isolated from *Amphora sp*. diatom was integrated into the calcium-magnesium-silicate-phosphate containing CB structure to develop BCB and the morphological, chemical, mechanical, thermal, and biodegradation properties of the CB and BCB materials were analyzed. Then, the effects of both scaffolds on cell biocompatibility and osteogenic differentiation capacity were assessed using mesenchymal stem cells (MSCs) derived from the bone marrow of rats. A comprehensive set of analyses, including alamar blue assay, alkaline phosphatase (ALP) activity, *alizarin red S* staining, reverse transcription-quantitative polymerase chain reaction (RT-qPCR), scanning electron microscopy (SEM), and micro computed tomography (micro-CT) were performed to show the effect of scaffolds on the cell viability and osteogenic differentiation. To the best of our knowledge, this study represents the first endeavor to develop a novel bioactive material by integrating diatom BS into the CB structure for applications in bone tissue engineering. Furthermore, the study assesses the suitability of BCBs and CBs as bone scaffolds for the first time.

## Materials and methods

**Figure 1:**
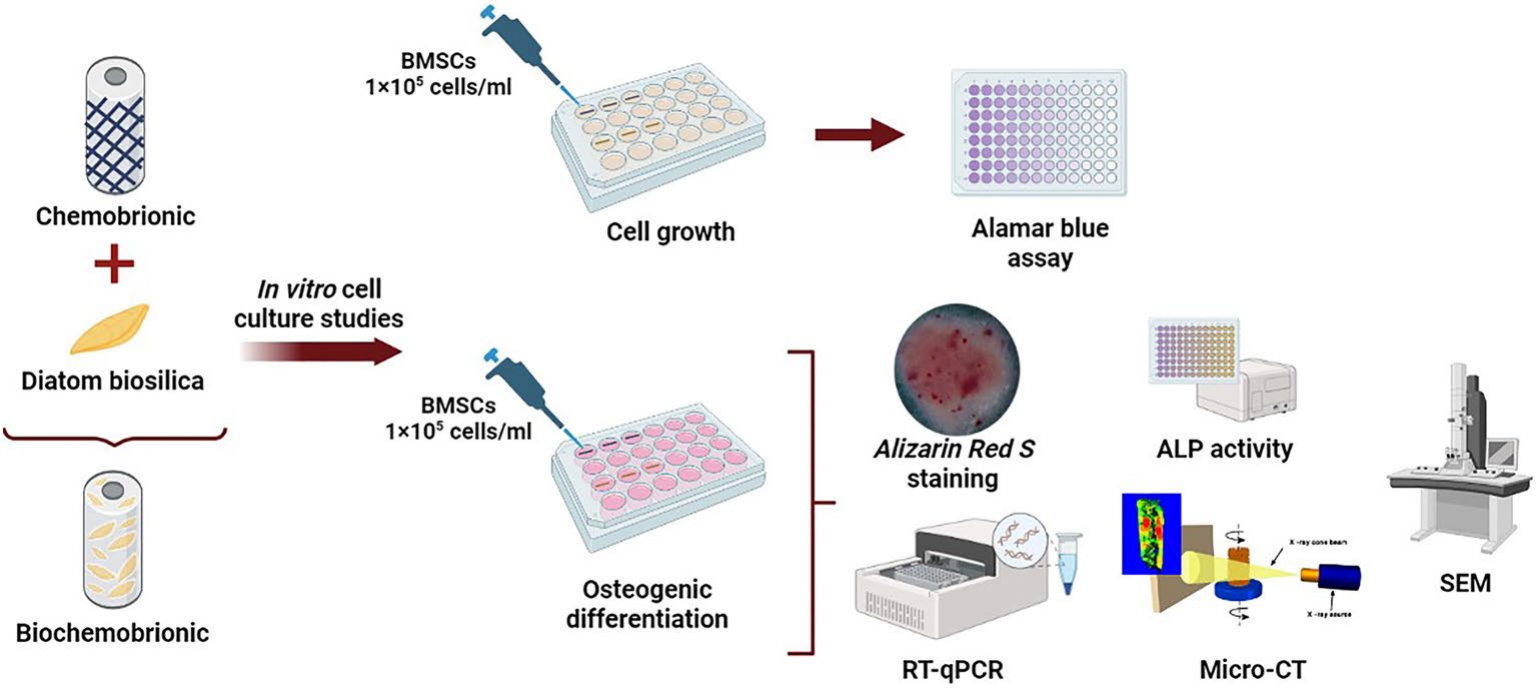
Schematic representation of experimental design.

A general shematic presentation of the experimental design and applied methods was illustrated in Fig. 1.

### Materials

*Amphora sp*. (CCAP 1001/3) was supplied from Culture Collection of Algae & Protozoa (CCAP) of The Institute of Freshwater Ecology, Cumbria, UK. Calcium chloride (CaCl_2_), magnesium chloride (MgCl_2_), hydrochloric acid (HCl) (37.0%) were the products of Carlo Erba (Milan, Italy). Sodium silicate (Na_2_SiO_3_), potassium phosphate (K_2_HPO_4_) were purchased from Sigma Aldrich (USA). For in vitro cell cultures and analyses, alpha modified Dulbecco’s Eagle’s medium (Alpha-MEM) (Capricorn, MEMA-XRXA), Dulbecco’s Modified Eagle Medium–High Glucose (DMEM-HG) (Capricorn, DMEM-HPA), fetal bovine serum (FBS) (Capricorn, FBA-11A), L-glutamine (Capricorn, GLN-B), penisilin-streptomycine (Capricorn, PS-B), ascorbic acid (Sigma, A8960), β-glycerophosphate (Sigma, G9891), dexamethasone (Sigma, D2915), phosphate-buffered saline (PBS) (Sigma, 12,636), trypsin–EDTA (Capricorn, L1805), alamar blue dye (Sigma, B7017-16), ALP activity assay kit (Abcam, ab83369), *alizarin red S* dye (Applichem, A2306), paraformaldehyde (Sigma, P6148), sodium cacodylate (Sigma, C0250), glutaraldehyde (Merck, S6088703 028), sucrose (Merck), osmium tetroxide (EMS, 1900), TriPure isolation reagent (Roche Diagnostics), mRNA isolation kit (Qiagen, 74,104), cDNA synthesis kit (Thermo Scientific, K1621) were used.

### Preparation of BS from diatom

The diatom *Amphora* sp. was cultured in sterilized F/2 Guillard medium ^24^ with a salinity of 30‰ and a silica content of 0.2 mM in a 10-L bottle at 24 ± 2 °C. The culture was maintained with constant aeration of 0.5 vvm under a light intensity of 65 µEm^−2^ s^−1^ on a 12:12 h light: dark cycle. The cells were harvested by sedimentation and centrifugation on the 12^th^ day of cultivation, washed with distilled water, and the harvested biomass was lyophilized.

To purify the BS, 50 mg of lyophilized biomass was treated with 50 mL of 15.0% (v/v) HCl at 95 °C for 2 h with continuous stirring. After the treatment, the frustule was collected by centrifugation at 2500 × *g* for 15 min. It was then washed with water 3–4 times to neutralize any residual acid. Then, an oxidation step was performed by adding 50 mL of 30.0% (v/v %) H_2_O_2_ to the frustule and stirring at 100 rpm at room temperature for 12 h. It was followed by centrifugation (2500 × *g*, 10 min) and washing with distilled water. Finally, the frustule was dried at 120 °C for 6 h, and subsequently stored in a desiccator until use ^25,26^.

### Production of CB and BS incorporated BCB scaffolds

CB and BS incorporated BCB scaffolds were produced with the controlled injection method ^27^. The system consisted of a New Era-100 syringe pump (USA) and a capillary tube to feed solutions, and a polypropylene centrifuge tube containing a hollow agarose template, which was 3.0 cm in height and had an internal space with a 3.0 mm diameter. To prepare the injection solution of BCB scaffold, 0.48 g MgCl_2_ (0.5 M in the final solution), 0.55 g CaCl_2_ (0.5 M in the final solution), and 0.2 g (2.0% w/v, which was determined in a preliminary experiment) BS powder were ground together in a mortar. Then, 10 mL of distilled water was added to the grinded mixture and mixed for 30 s with vortex. To disperse the frustule completely, the suspension was sonicated in an ultrasonic bath (Hydra Ultrasonic, Turkey) for 15 min and its pH was set to 2.5 by using 1.0 N HCl. This solution was fed into the alkaline solution (pH = 13.0) containing 3.0 M Na_2_SiO_3_ and 0.5 M K_2_HPO_4_ by using a syringe pump through a 0.5 mm internal diameter needle with a constant injection rate of 4 mL/h. In order to avoid the sedimentation or collapse of BS, the injection solution was continuously stirred by a magnetic stirrer during the process. For the production of CB scaffold, the injection solution of 0.5 M CaCl_2_ and 0.5 M MgCl_2_ was injected into the alkaline solution of 3.0 M Na_2_SiO_3_ and 0.5 M K_2_HPO_4_ with an injection rate of 2 mL/h. The growth of CB and BCB scaffolds was performed for 2 h, obtained structures were collected from the solution, washed with distilled water, and dried at room temperature in a desiccator.

### Characterization of scaffolds

#### Morphology analysis

The micro morphologies of the CB and BCB scaffolds were observed by SEM (Thermo Scientific Apreo S Instrument, ThermoFisher Scientific, USA). Prior to imaging, samples with standard dimensions (0.3 mm × 10 mm; diameter × height) were gold coated using a sputter coater.

#### Chemical analysis

To identify the chemical bonds present in the scaffolds, attenuated total reflectance-Fourier transform infrared spectroscopy (ATR-FTIR) was performed. Analysis was conducted with Spectrum Two spectrophotometer (Perkin Elmer, USA) in the range of 500–4000 cm^−1^ using the KBr technique^28^.

#### Thermal analysis

Thermogravimetric analysis (TGA) was carried out to investigate thermal stabilities of the scaffolds. Analysis was performed between 25 to 800 °C at a heating rate of 10 °C min^–1^ under nitrogen atmosphere.

#### Mechanical analysis

Mechanical characteristics of the produced scaffolds were analyzed by using uniaxial compressing test with a mechanical testing device (Hybrid Rheometer Discovery HR-2, TA Instruments, USA). At least three different samples (3.5 mm width and 5.0 mm length) were tested at a rate of 6.0 mm/min until failure. Stress–strain curves were plotted from the obtained data and compressive modulus was determined from this slope.

#### Degradation analysis

The solution-mediated degradations of scaffolds were evaluated using a simulated body fluid (SBF) solution. The samples were completely immersed in 10 mL of SBF solution, which was prepared as described in detail elsewhere ^20^. Immersed samples were incubated at 37.0 ± 1.0 °C for different time periods up to 28 days, washed with distilled water at the end of the periods, and dried at room temperature. The degradation rate of scaffold was calculated based on the following Eq. (1) by measuring the weight of samples before (W_0_) and after (W_t_) immersion in the SBF at time t.

1 $$\Delta {W}_{t}=\frac{{W}_{0}-{W}_{t}}{{W}_{0}}$$

### In vitro cell culture studies

#### Cell culture

The MSCs derived from the bone marrow of rats were provided from Ege University Bioengineering Department Biomaterials and 3D Biointerphases Laboratory (EBIOPHASE) collection and used with the permission of the Ege University Animal Experiments Local Ethics Committee (EUHADYEK) numbered 2015–020 and dated 25.02.2015. 200–250 g 12 ± 2 months old 2 male adult Wistar-Albino rat bone MSCs were pooled and used for both cell proliferation and differentiation analyses.

MSCs were cultured with α-MEM medium containing 10% (v/v) FBS, 0.1% (v/v) penicillin–streptomycin, and 1.0% (v/v) L-glutamine in a humidified incubator at 37 °C containing 5% CO_2_. Once cells reached 80% confluency, they were passaged. Prior to in vitro experiments, scaffolds were sterilized with ethylene oxide steam for 24 h at room temperature, followed by aeration for 24 h to eliminate the gas retained. Then, they were placed in 24-well culture plates, and they were conditioned with α-MEM medium containing 10% (v/v) FBS, 1.0% (v/v) L-glutamine, and 0.1% (v/v) penicillin–streptomycin for 2 h. MSCs were seeded at a concentration of 1 × 10^5^ cells/ml onto the surface of scaffolds. The incubation was carried out under standard conditions for 28 days by changing the medium every two days. The average dimensions of the scaffolds used are height (h) 10 mm, radius (r) 1.5 mm, surface area (A) 108 mm^2^ and volume (V) 70 mm^3^. The medium used is 500 µL for 24 well.

For osteogenic differentiation, MSCs were first cultured for 48 h in α-MEM medium containing 10% (v/v) FBS, 0.1% (v/v) penicillin–streptomycin, and 1.0% (v/v) L-glutamine. Then, growth medium (GM) was removed from each well, and osteogenic differentiation medium (ODM), composed of DMEM-HG with 10% FBS, 0.1% (v/v) penicillin–streptomycin, 200 μM ascorbic acid, 10 mM beta-glycerophosphate, and 100 nM dexamethasone, was added to the scaffolds. The culture media were changed every 2 days, and the cultivation was carried out for 28 days in a 5% CO_2_ atmosphere at 37 °C.

#### Alamar blue assay

The viability of MSCs was assessed using the alamar blue assay at different time points, including 1, 4, 7, 14, and 21 days of culture. At these days, the culture medium was replaced with fresh α-MEM containing 10% alamar blue dye. The cells were then incubated at a temperature of 37 °C for a duration of 4 h. After incubation, 100 µL of medium was transferred to a 96-well plate, and optical density of the media were measured at 570 nm and 600 nm using a microplate reader (Synergy HTX- BioTek Instruments, USA).

#### *Alizarin red S* staining

The calcium deposition of the MSCs cultured on scaffolds was evaluated by alizarin red staining after 28 days of incubation. Culture media were removed from culture wells, and scaffolds were washed with Ca^+2^-Mg^+2^ free PBS three times. Cells were fixed with 4.0% paraformaldehyde for 30 min and washed with PBS again. Then, scaffolds were stained with a 2.0% alizarin red (pH 4.1–4.3) solution for 2–3 min, and after that, they were washed with PBS many times till the PBS remained colorless. To capture the images of stained scaffolds, an inverted phase-contrast microscope (Olympus, Japan) was used. The calcium deposition on the scaffolds was also examined using the colorimetric analysis of alizarin red staining samples. After monitoring, stained scaffolds were treated with a solution of 20% methanol and 10% acetic acid in water for 15 min under shaking conditions. Then, the liquids with dissolved calcium were transferred to 96-well plates, and their optical densities were measured at 450 nm with a microplate reader ^29^.

#### ALP activity

To measure the level of ALP activity, a colorimetric assay kit (Abcam Inc., USA) was used after 21 and 28 days of culture, according to the manufacturer’s instructions. Briefly, an 80 µL cell lysate solution was transferred to a 96-well plate, and 50 µL of the 5 mM *p*-nitrophenyl phosphate (pNPP) solution was added to each well. They were mixed, and the reaction was carried out at 25 °C under dark conditions for 1 h. At the end of the period, 20 µL of a stop solution was added to each well, and the absorbance of the samples was measured at a wavelength of 405 nm using a microplate reader. The ALP activity (U/ml) of each sample was determined according to the calibration curve of p-nitrophenol standards.

#### RT-qPCR

The expression levels of osteogenic-specific genes, including ALP, osteocalcin (OCN), osteopontin (OPN), and osteonectin (ON) were determined on day 28 by RT-qPCR. Primer sequences were determined with the web based Primer3 program. The suitability of the primers was confirmed with a web-based in silico PCR program. β-actin was preferred as the internal standard. The primers were commercially synthesized ^30,31^. Total mRNA of MSCs was extracted using TriPure isolation reagent (Roche Diagnostics) and RNeasy mini kit (Qiagen, CA, USA). Briefly, the medium was removed from culture wells, and scaffolds were washed twice with Ca^+2^ -Mg^+2^ free PBS. 1 ml of chilled TRIzol Reagent (Invitrogen, USA) was added to each well, scaffolds were disrupted with pipetting, and they were transferred to the RNase-free tube, followed by the incubation in TRIzol for 5 min at 20 °C. Then, 200 µL of chloroform was added to tubes, the mixture was vortexed, incubated for 15 min at 20 °C, and centrifuged at 4 °C for 15 min at 12,000 *g*. The upper aqueous phase was transferred to an RNase-free tube and same amount of 70% ethanol was added ^32^. After carefully mixing, 700 µL of the mixture was transferred to a spin column, and RNA extraction was carried out according to the manufacturer’s instructions. To reverse-transcribe the extracted RNA into cDNA, Revert Aid First Strand cDNA synthesis kit (Thermo Scientific) was used. Real-time PCR was conducted using 2X qPCR SYBR-Green MasterMix reagents (Applied Biosystems, USA). Primers used for bone regeneration, OCN, OPN, ON, and ALP, are presented in Table S1. The relative transcript quantities were determined using the ΔΔCt method, where β-actin was used as the reference gene ^33^.

#### Cell morphology and chemical composition

After 28 days of incubation, SEM–EDS analysis was carried out to assess cellular morphological features and chemical composition of the scaffolds’ surface. After washing the scaffolds with PBS (4 °C, pH = 7.0), the cells were fixed for 30 min in 5% (v/v) glutaraldehyde in a 0.1 M sodium cacodylate solution, followed by 7.0% sucrose in a 1.0 M sodium cacodylate solution. A post-fixation step was performed with 2.0% osmium tetroxide in a 0.1 M sodium cacodylate solution at room temperature for 1 h. Scaffolds were washed with distilled water and dehydrated in a graded series of ethanol solutions ranging from 35 to 100% (v/v) and in hexamethyldisilazane. Finally, scaffolds were coated with gold under vacuum conditions and observed by SEM ^34^. EDS analysis was performed using an equipment coupled with SEM. To ensure the representativeness of the EDS analysis, at least three spectra were acquired from each specimen with random selection.

#### Micro-CT

For the determination of mineralization in the scaffolds, a micro-CT analysis was performed using the Scanco Medical μCT50 (Switzerland). The scaffolds were scanned before sterilization, and they were incubated under the aforementioned differentiation conditions. After a 28-day incubation period, the samples were washed with PBS and then fixed with a 4% paraformaldehyde solution at room temperature. The scaffolds were carefully positioned in the sample holder of the device and scanned with the following settings: 70 kV, 114 μA source current, 300 ms integration time, 5 μm voxel size, and 360° rotation range. Corresponding 3D images from the obtained 2D slices were reconstructed using micro-CT assistant software.

### Data analysis

All the experimental groups were carried out at least in triplicate and the results were presented as mean ± standard deviation (SD). After having verified normal distribution and homogeneity of variance, one-way or two-way analysis of variance (ANOVA) with Tukey or Dunnett's multiple tests were used to test for statistically significant differences for comparisons. Statistically significant values were defined as ns: p > 0.05, **p < 0.01, ****p < 0.0001.

## Results and discussion

### Characterization of CB and BCB scaffolds

#### Micromorphology

**Figure 2:**
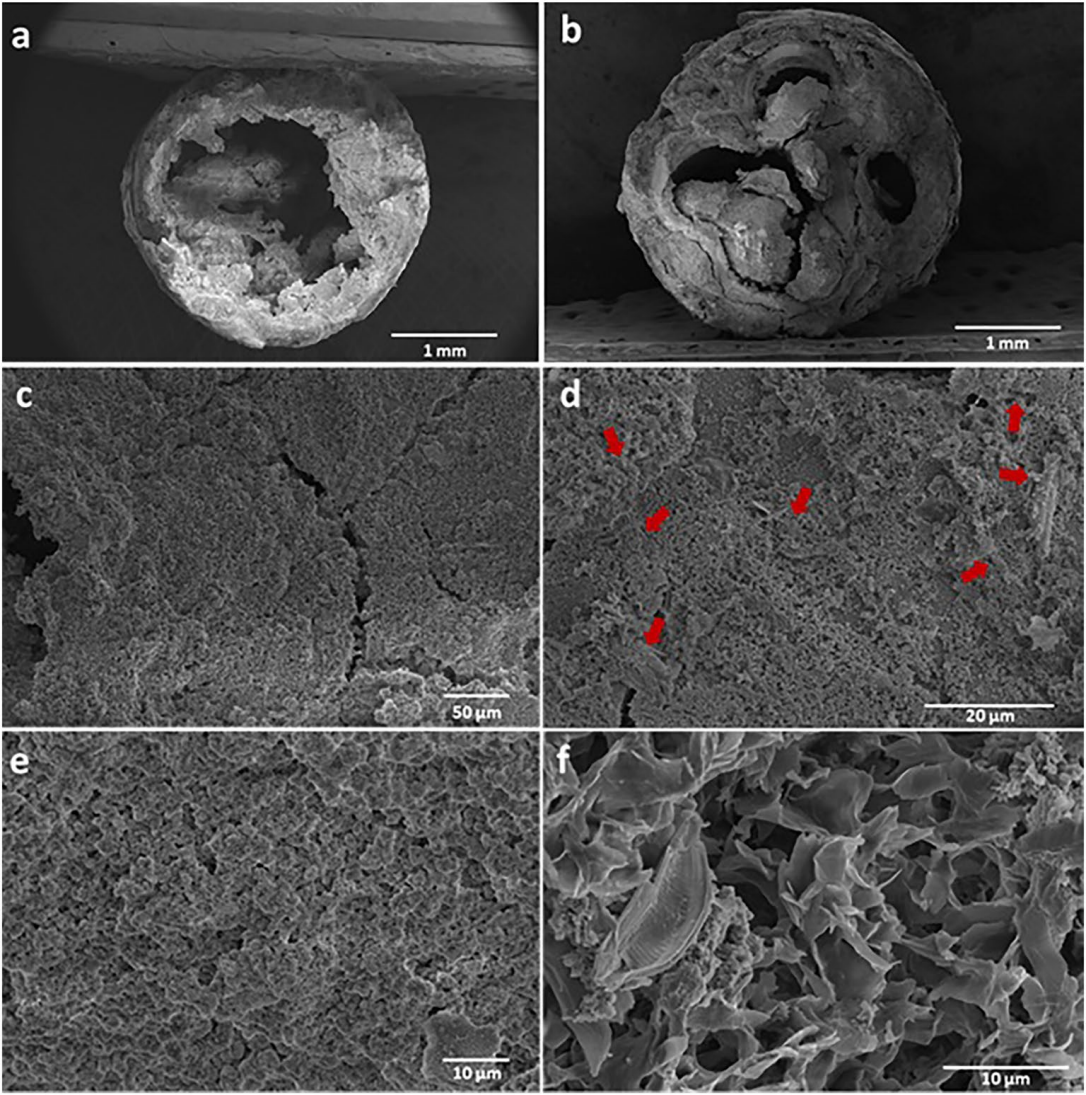
SEM micrographs of CB and BCB scaffolds; cross-sectional area of (**a**) CB, (**b**) BCB, exterior surface of (**c**) CB, (**d**) BCB, interior surface of (**e**) CB, (**f**) BCB.

The morphologies of the CB and BCB scaffolds are presented in Fig. 2. The cross-sectional appearance of samples showed that they had different morphologies. CB had a hollow tubular structure (Fig. 2a), while BCB exhibited precipitate deposition in its interior volume, which resulted in the formation of an irregular porous structure that exhibited macro-sized (up to 500 µm) and randomly distributed pores (Fig. 2b). Previous studies of CB systems via SEM analysis have reported that the tubes have hollow structures with membrane-like walls of varying thickness ^18,35,36^, which confirmed the obtained morphology of CB (Fig. 2a). In this study, the integration of diatom BS led to changes in the growth pattern of BCB and resulted in the formation of a non-hollow tubular structure whose interior space had dense precipitate deposition. The growth behavior and resulting morphology of the obtained BCB enabled the development of a more suitable material for use as a scaffold due to its porous structure and high inorganic mineral accumulation. The interior and exterior surfaces of the scaffolds had a rough surface with particles of different sizes and microcracks, which might be formed during fabrication, extraction, or drying steps (Figs. 2c–f). SEM images at higher magnification indicated that the BS was embedded on the exterior surface of the structure (Fig. 2d-red arrows), while its integration into the interior space was achieved more effectively (Fig. 2e). According to the overall morphology, BS was distributed randomly and homogenously. It has been observed that a highly porous structure consisting of micro sized pores was obtained in the areas where the amount of integrated BS was increased. These pores increase the material's surface area, resulting in the induction of mineralization and osteogenesis through protein adsorption, ion exchange, and osteoblast differentiation ^7,37^. The nanopores found in BS surface were blocked by the inorganic precipitates derived from the reaction of metal salts and anions (Fig. 2e).

#### Chemical composition

**Figure 3:**
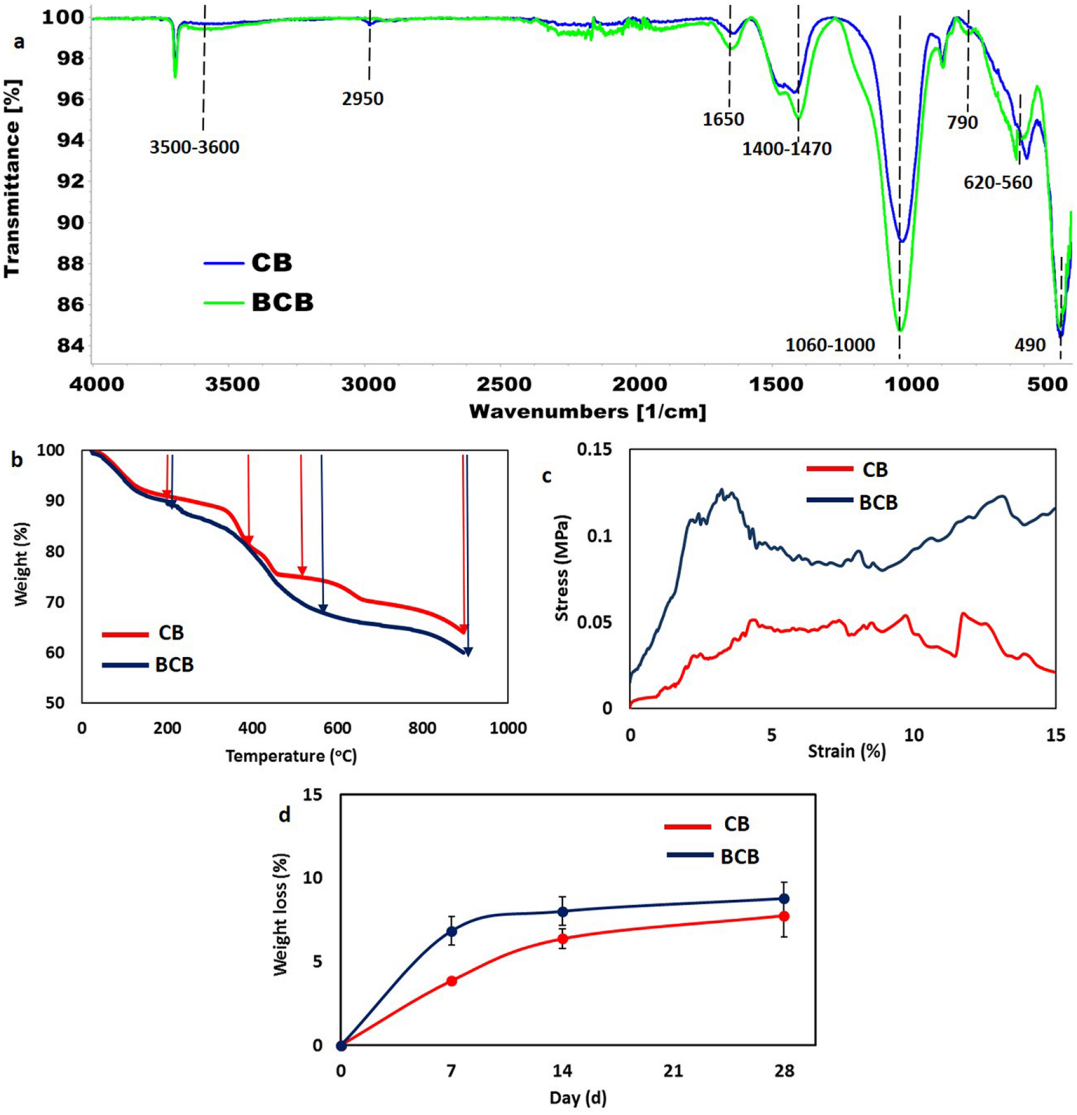
(**a**) FTIR spectra of the CB and BCB scaffolds, (**b**) Thermal properties, (**c**) Stress–strain curves in compression test, and (**d**) In vitro degradation test of CB (red) and BCB scaffolds (blue).

CB and BCB scaffolds were subjected to FTIR analysis to determine their chemical compositions (Fig. 3a). According to literature, the FTIR spectrum of purified BS has a main peak at 1061 cm^−1^, which is attributed to asymmetric stretching vibrations of Si–O–Si bonds. The peak located at 940 cm^−1^ is ascribed to the Si–OH stretching vibration for silanol groups. In addition, the band at 790 cm^−1^ is assigned to symmetric stretching vibrations of Si–O–Si ^38,39^. The broad band at 3500 cm^–1^ is due to O–H bonds in silanol groups or H–O-H bonds of molecular water. Besides, the bands at 2940 cm^−1^ and 1742 cm^−1^ are related to C–H and C=O bands, respectively, which indicates the presence of residual organic materials in frustule ^40^. CB scaffold showed several bands at 1640 cm^−1^, 1500–1400 cm^−1^, 1020 cm^−1^, 870 cm^−1^, 580 cm^−1^, and 440 cm^−1^, which were associated with the O–H, C–O, Si–O–Si, P–O, and Mg–O/Ca–O bonds, respectively. In the spectrum of the BCB scaffold, the intensity of the peak located at 1020 cm^−1^ increased and shifted to 1040 cm^−1^ due to integrated silica. In addition, there were new band formations or small increases in the existing bands at 3500 cm^−1^, 2920 cm^−1^, 1650 cm^−1^, 1460 cm^−1^, 790 cm^−1^, and 620 cm^−1^. These results confirmed that the purified BS from the diatom was successfully integrated into the BCB scaffold.

#### Thermal and mechanical properties

TGA was performed to determine the thermal stability and degradation behavior of the scaffolds with respect to temperature. For both scaffolds, an initial weight loss of ~ 9% was recorded between 0 and 200 °C, which was due to the vaporization of physically bound water from the samples (Fig. 3b). In the thermogram of CB, a step-wise weight loss was observed between 200 and 600 °C, which was due to the loss of Mg-O and Ca-O. BCB scaffold also showed a similar but higher amount of weight loss in this range because of the thermal decomposition of residual organics in diatom BS. The last stage, starting at ~ 600 °C and completing at 900 °C, was the dehydroxylation of silanol hydroxyl groups. This change was observed in both scaffolds since silica was one of the main components of both scaffolds ^39,41^. However, the total weight loss of the BCB scaffold (39.89%) was higher than that obtained in the CB scaffold (35.93%) due to the thermal decomposition behavior of the integrated BS.

One of the major challenges of CB systems is their poor mechanical performance due to their fragile nature, which makes the characterization and application of these structures difficult. Analyzing and improving the mechanical properties of CBs have been reported as highly challenging for further research ^14,42^. In this study, a compression test was applied to CB and BCB scaffolds with a dimension of about 3.5 mm × 5.0 mm, and their stress–strain curves are presented in Fig. 3c. Both samples exhibited a multi-peak jagged profile, which had a positive slope up to a first peak, and then compressive stress began to drop because of starting cracks. At this point, microcracks found in the structure’s surface and disordered macroscopic pores located inside the samples affected the mechanical behavior of the structure. However, the samples were still able to bear ongoing loads due to the densification of the fractured precipitates under compression. Therefore, the stress rose again, and repetition of this process led to the formation of a jagged stress–strain curve. The resulting stress–strain diagrams of both samples showed a typical brittle curve that is representative of those generally obtained with ceramic and glass materials ^43^. When the effect of BS was examined in BCB, the CB scaffold showed a maximum compressive strength of 0.065 ± 0.006 MPa, while this value increased to 0.14 ± 0.02 MPa for BCB, which was significantly different between two groups (p < 0.01). This result demonstrated a twofold increase in compressive modulus, due to the existence of BS (Fig. 3c), which was consistent with previous studies reporting that the addition of diatom BS improved the mechanical properties of scaffolds in terms of compressive or Young’s modulus ^6,7,44^. Scaffolds for repairing bone defects should possess good mechanical properties, ideally matching or exceeding those of the surrounding bone to reconstruct hard load-bearing tissues. While the results of the compression tests demonstrated improved mechanical strength with the integration of BS into the CB structure, it's noteworthy that the maximum compressive strength obtained was still lower than that of trabecular and cortical bones^45^. In this context, the BCB material may be a suitable candidate for structural support or as a bone void filler, particularly in applications where superior mechanical strength is not a primary requirement. For instance, Hughes et al. ^22^ developed a bone-augmenting material composed of calcium-loaded hydrogel-based spheres. These spheres were placed in a cylindrical bone defect introduced into an ex vivo human tissue sample. Upon exposure of the defect to a phosphate-rich medium, hollow white precipitate tubes formed, exhibiting similar properties to CB material. This demonstrates the applicability of such materials as bone void fillers.

#### In vitro biodegradation analysis

Fabricated CB and BCB scaffolds were immersed individually in SBF for 7, 14, and 28 days to examine the degradation rates by measuring their weight loss (Fig. 3d). The CB sample underwent a weight loss of 3.86 ± 0.05% during the first 7 days, while the weight loss of the BCB scaffold during this period was 6.84 ± 0.84%, which was significantly different between two groups (p < 0.05). At day 14, there was no significant difference in the degradation rate, with weight losses reaching 6.37 ± 0.57% and 9.01 ± 0.85% for CB and BCB, respectively. Finally, the weight loss of the BS incorporated BCB scaffold was 1.7-fold higher than that of the CB scaffold. The increase in degradation rate of the BCB scaffold was attributed to the incorporated diatom silica, which was proven to have a natural degradable behavior in previous studies ^46,47^. The differences in the weight loss of the samples showed that the CB and BCB scaffolds may take different time intervals for complete degradation in vitro. The biodegradable materials play a significant role in repairing bone defects. Ideally, these materials should be mechanically stable until the formation of new functional tissue, yet degrade sufficiently to provide enough space for new tissue growth. Several critical factors in the design of bone graft materials, such as anatomical location, patient age, and type of injury, influence the appropriate degradation rate. Therefore, it is not possible to determine an optimum degradation rate that is universally valid for all bone tissue scaffolds ^48,49^. The different degradation rates of CB and BCB materials indicated that they can be utilized in different cases, considering the mentioned critical factors.

### In vitro cell viability

**Figure 4:**
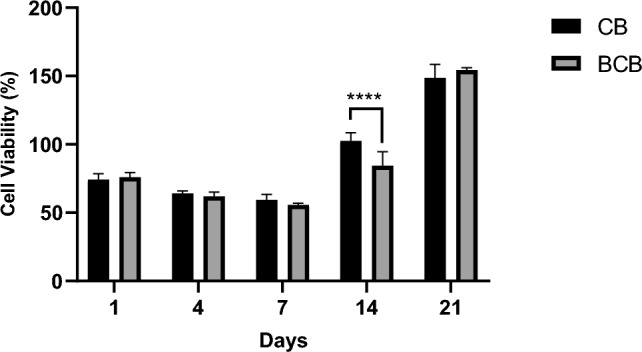
Comparison of alamar blue analysis of CB and BCB scaffolds on days 1, 4, 7, 14 and 21 (****p < 0.0001).

Cell viability on fabricated scaffolds was analyzed at 1, 4, 7, 14, and 21 days of incubation using the alamar blue assay (Fig. 4). It was observed that cells adhered to both CB and BCB scaffolds and showed metabolic activity over time. Initially, cell viability of more than 50% was observed in both samples on day 1. However, during the adaptation period between days 1 and 7, the number of cells tended to decrease. After this adaptation period, metabolic activity increased, leading to significantly increased cell proliferation in both groups at day 14 and day 21. Interestingly, the viability on the CB scaffolds was significantly higher than that on the BCB scaffolds at day 14, although it was almost equal at day 21. Overall, no significant difference was found between CB and BCB scaffolds in terms of cell viability. These results suggest that both scaffolds did not elicit any cytotoxic effect; on the contrary, they supported the proliferation of MSCs. In previous studies examining the CB-cell interaction, the effects of calcium and phosphate-containing structures on cell viability were generally observed. For this purpose, in vitro culture studies were carried out with the cancer cell line HeLa and sheep MSCs. These studies stated that the CB surface did not create any cytotoxic effect in terms of cells and had growth and proliferation-supporting properties ^21,23^.

### Osteogenic differentiation in vitro

**Figure 5:**
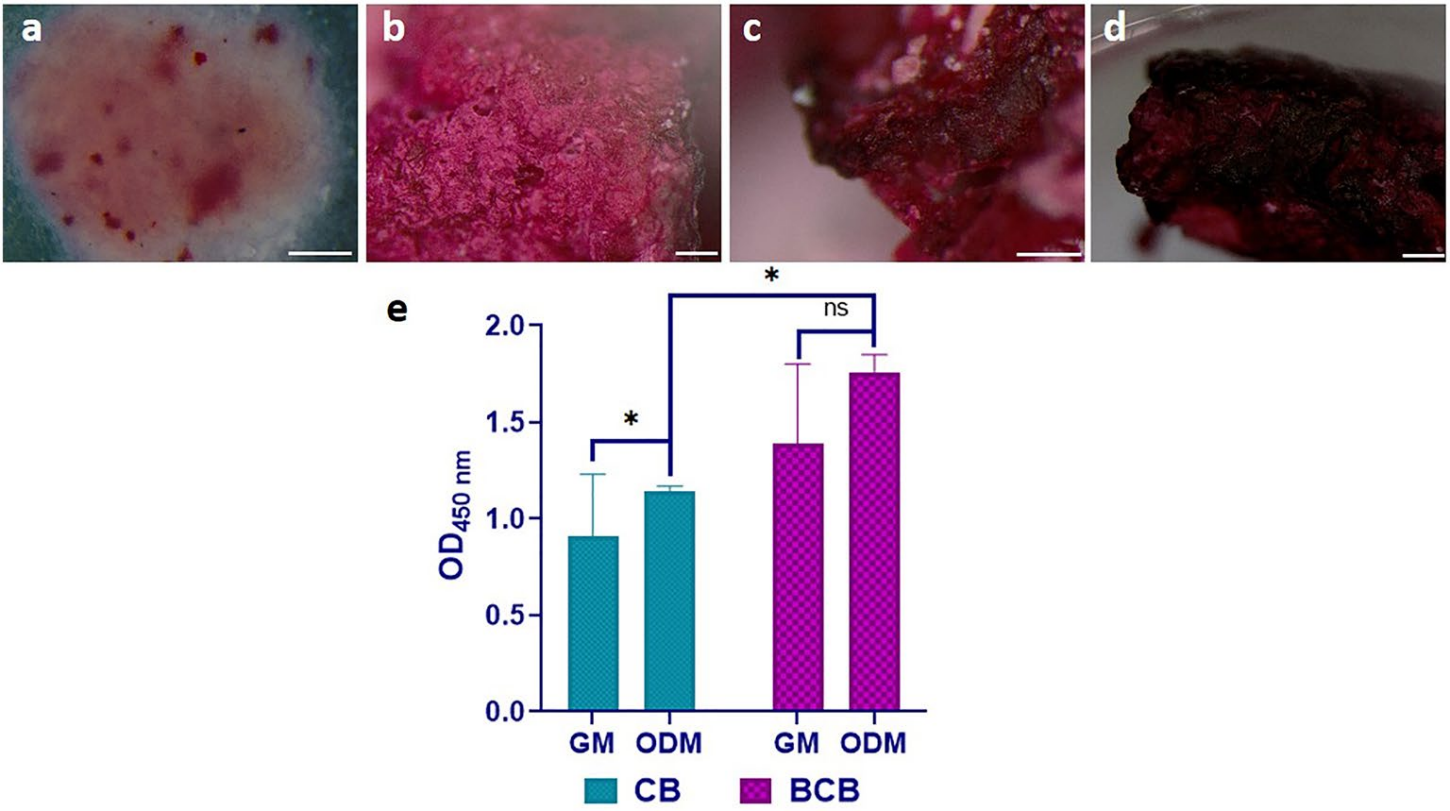
*Alizarin red S* staining of (**a**) MSCs cultured in ODM on the surface of well-plate (Scale bar: 20 µm), (**b**) blank (cell-free) BCB scaffold (Scale bar: 200 µm), (**c**) MSCs cultured in ODM on CB scaffold (Scale bar: 200 µm), (**d**) MSCs cultured in ODM on BCB scaffold (Scale bar: 500 µm) (**e**) Quantitative analysis of *alizarin red S* staining of CB and BCB scaffolds in GM and ODM after 28 days of incubation (ns; p > 0.05, *p < 0.05).

*Alizarin red S* is an important marker that is based on the staining of calcium deposition generated from cells during osteogenic mineralization ^50^. After 28 days of incubation, osteogenic differentiation was determined by using *alizarin red S* staining, and obtained results are presented in Fig. 5. In order to confirm the differentiation capability of MSCs, cells were firstly incubated on the surface of the well plates in ODM, and the calcium deposits on the plate surface were clearly visualized by *alizarin red S* staining (Fig. 5a). When comparing the mineral deposition on CB and BCB scaffolds, the observations were highly difficult because the chemical composition of the scaffolds already consisted of a high amount of calcium, which turned red by staining without any cellular interaction or osteogenic differentiation (Fig. 5b). Despite this challenge, the microscopic images clearly showed that the BCB scaffold had a considerable increase in dense red-colored areas compared to those obtained in the CB scaffold (Figs. 5c,d). To further confirm the difference between CB and BCB, a semi-quantitative colorimetric assay was performed considering the absorbed alizarin red amounts into the scaffolds (Fig. 5e). There was an obvious increase in the mineral deposition level of both scaffolds incubated in ODM compared with those incubated in GM, but these differences were not statistically significant (p > 0.05). The higher level of mineralization was an expected result since the CB scaffold had great potential to mimic the bone structure in terms of its chemical composition, including Ca, Mg, Si, and P. Despite the promising role of CBs in bone tissue applications, a detailed investigation of the in vitro behavior of these structures for osteogenic differentiation is less explored. In a study conducted by Hughes et al. ^22^, a bone augmenting material was developed by the precipitation reaction of calcium and phosphate. They showed the formation of calcified nodules by performing *alizarin red S* staining and thus reported the osteoconductive characteristics of tubular structures consistent with clinically applied calcium phosphate. Considering the effect of BS incorporation into the CB, the amount of mineralized matrix production was significantly higher in the BCB scaffold than in the CB (p < 0.01) (Fig. 5e). These results indicated that the presence of diatom BS enhanced the mineralization ability of BCB scaffold. Many previous studies conducted *alizarin red S* staining to detect the osteogenic differentiation and reported that the addition of diatom BS to scaffolds increased the intensity of calcium nodules and thus the degree of mineralization ^6,51–53^.

**Figure 6:**
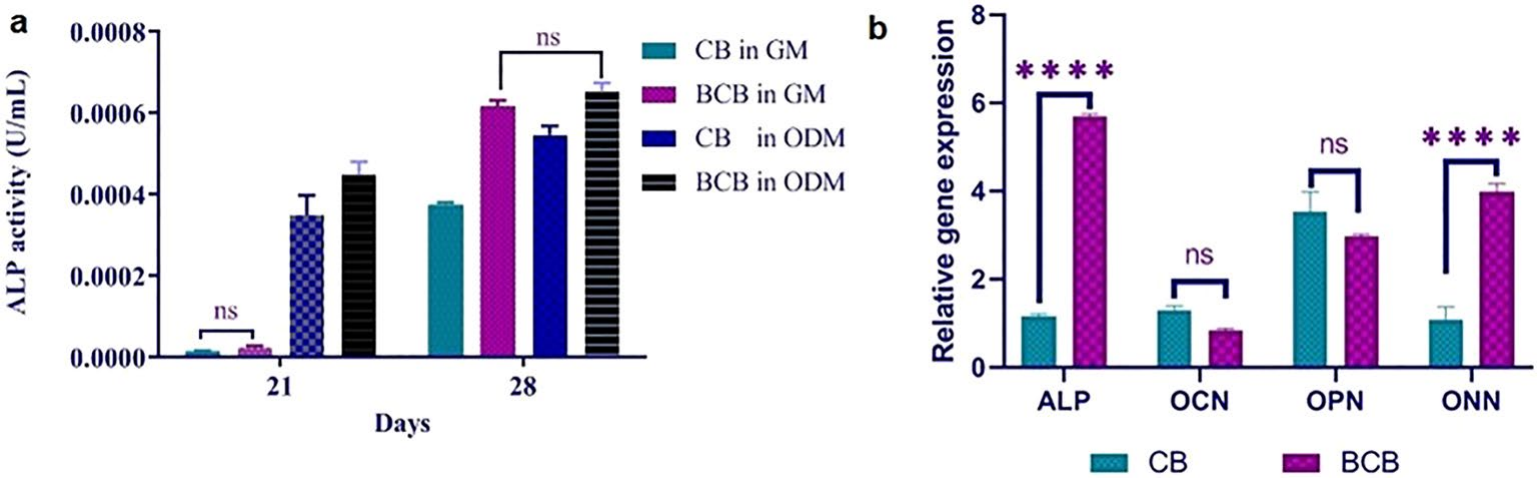
(**a**) ALP activities of MSCs cultured on scaffolds in GM and ODM for 21 and 28 days, (**b**) Gene expression levels of ALP, OCN, OPN, and ON of MSCs culture on CB and BCB scaffolds in ODM.

Another common marker used for osteogenic differentiation is ALP, which is a byproduct of osteoblast activity. The increase in the amount of ALP in the osteogenic differentiation process of MSCs suggests active bone formation ^54^. In this study, it was measured for cells incubated in GM and ODM for CB and BCB scaffolds after 21 and 28 days (Fig. 6a). At day 21, the ALP activities of MSCs on both scaffolds were higher in the ODM than in the GM. At day 28, the ALP activities of MSCs on CB scaffolds both in GM and ODM increased and reached the values of 0.0003 U/mL and 0.0005 U/mL, respectively. It has been known that scaffolds containing Ca and P induce high ALP levels during osteogenic differentiation of stem cells ^55–57^. Compared to the MSCs on CB scaffolds, MSCs on BCB scaffolds had significantly higher ALP activities, which were 1.36-fold and 1.24-fold higher than the activities of MSCs on CB scaffolds at 21 and 28 days of cultivation, respectively. Overall, BCB scaffold provided higher osteogenic differentiation than CB scaffold because of the osteogenic activity of the incorporated diatom BS. The positive effect of diatom BS on ALP activity could be a result of the induction of osteoblastic activity by the soluble silicon and stimulation of the osteoblast response by affecting the level of differentiation markers for biomineralization ^3,6,58^. On the other hand, at day 28, a distinct trend was observed in ALP analysis; there was no significant difference in activity between the cells cultured on BCB scaffolds in GM and ODM. When considering alizarin red staining and ALP activity together, obtained results suggested that BCB scaffold induced osteogenic differentiation on cells even in the absence of osteogenic inducers like dexamethazone, ascorbic acid, β-glycerophosphate, etc. Previous studies have reported that calcium phosphate is a significant stimulator for mineralization during bone formation, and the scaffolds that include calcium and phosphate constituents are able to differentiate osteoblasts without osteogenic differentiation additives ^59–61^. Moreover, natural BS from diatom has been shown to induce the precipitation of calcium phosphate, stimulate osteoblast proliferation, and inhibit osteoclast formation ^8^. These outcomes revealed that BCB scaffold with calcium phosphate content can promote differentiation of MSCs in the absence of osteogenic differentiation supplements in the medium, and the incorporation of diatom BS further enhances the mineralization efficiency for bone formation.

The relative gene expression levels of the osteogenic markers, including ALP, OCN, ON, and OPN, in differentiated MSCs on CB and BCB scaffolds on day 28 were determined by RT-qPCR. The expressions of all markers were upregulated in different levels on both scaffolds (Fig. 6b). The relative mRNA level of ALP in cells on the BCB scaffold was found to be significantly increased compared to the cells on the CB scaffold (p < 0.001). Also, the relative gene expression of ON in differentiated MSCs on the BCB scaffold was higher than the cells on the CB scaffold (p < 0.001). There were no significant differences in OCN and OPN mRNA levels between cells on the scaffolds. In the case of differentiation, the expression of osteogenic specific genes, such as ALP, OCN, OPN, ON etc., is induced to regulate the differentiation stages of osteoblasts. ALP is a commonly used marker to assess early osteogenesis and plays a crucial role in initiating and enhancing mineralization ^62^. ON is a glycoprotein in the bone that is highly expressed at the beginning of differentiation by osteoblasts ^63^. Similarly, OCN and OPN are crucial markers for bone regeneration that belong to the late stage differentiation ^64^. The higher expression levels of ALP and ON genes on BCB scaffold showed that osteogenic differentiation was achieved more effectively than the CB scaffold, and osteogenic cells were at the early stages of differentiation. Also, the low expression degree in OCN and OPN markers and the non-significant difference between the two scaffolds were attributed to the fact that cells were at the early stages of differentiation, whereas these markers belong to the late stages.

**Figure 7:**
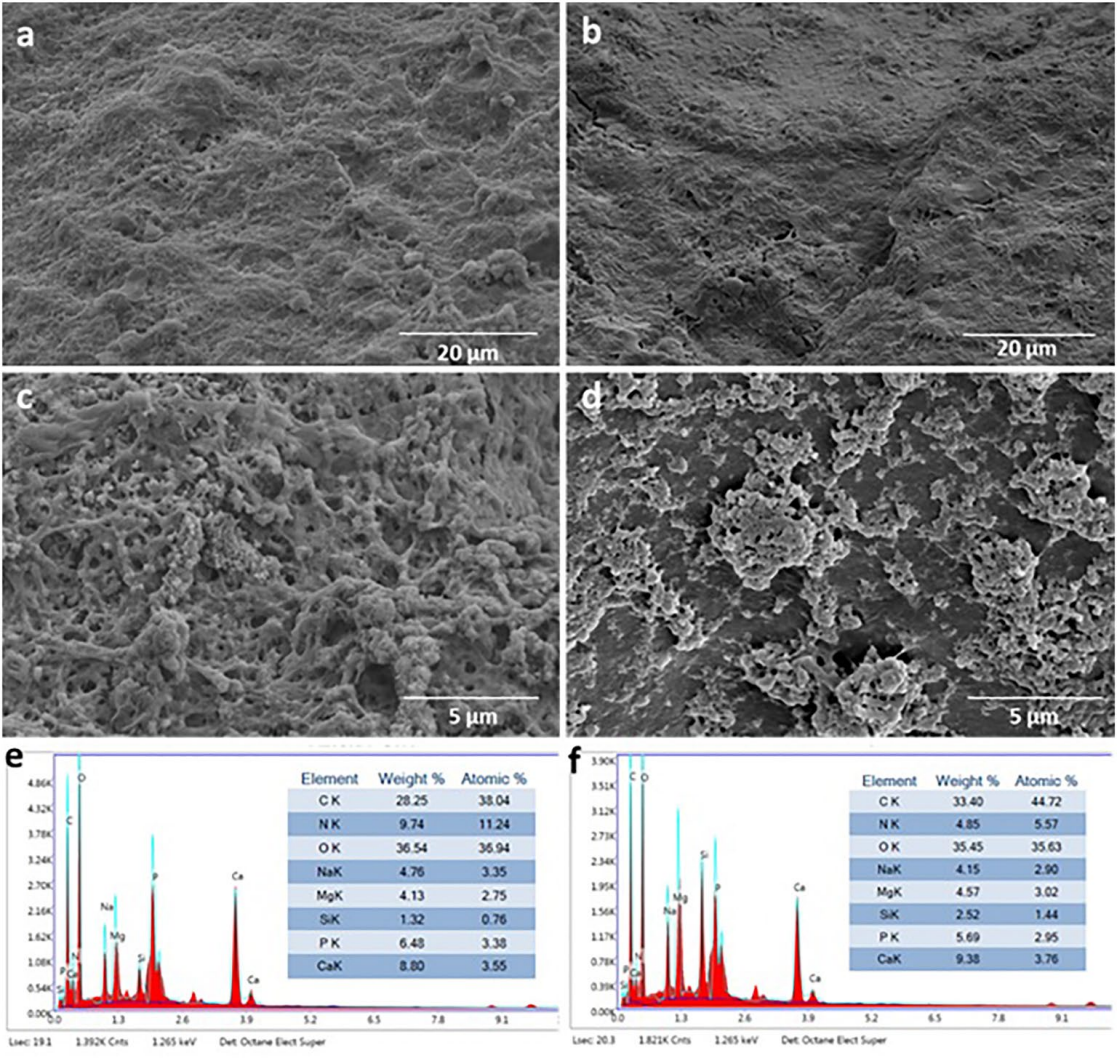
SEM images of MSCs after 28 days of incubation in ODM; (**a**,**c**) CB scaffold, (**b**,**d**) BCB scaffold. EDS analysis of MSCs after 28 days of incubation in ODM; (**e**) CB scaffold, (**f**) BCB scaffold.

In order to evaluate the morphology of MSCs cultured on CB and BCB scaffolds in differentiation medium and characterize the forming mineralization structures, SEM analysis was performed on the 28th day of incubation. As shown in Fig. 7, cells adhered and spread onto both scaffolds by covering the particulate surfaces. The porous structure of the scaffolds and incorporated diatom BS were no longer detectable, and abundant ECM formation was observed. Spherical particles, which indicated mineral deposition from cell differentiation, were observed in a broad area of BCB scaffold but also in a small proportion of the CB scaffold (Figs. S1 and S2). Punia et al.^21,65^ reported that different types of mammalian cells completely covered the surface of tubular CBs containing calcium, silica, and phosphate, and maintained their viability for a long time. However, up to now, the morphological changes of stem cells on CBs during osteogenic differentiation have been unexplored. The chemical compositions of the particles deposited on the scaffolds were characterized using EDS (Figs. 7e,f). The main aim of this analysis was to measure the Ca and P contents of particles because apatitic calcium phosphate is a substantial component in cortical bone with an ideal Ca/P ratio of 1.6 ^64^. The osteogenic differentiation in both scaffolds was further confirmed by EDS, with the stoichiometric Ca/P ratio being very close to 1.6 for CB (1.57 ± 0.31) and BCB (1.45 ± 0.27). However, there was no statistically significant difference between CB and BCB in terms of Ca/P ratio (p > 0.5).

**Figure 8:**
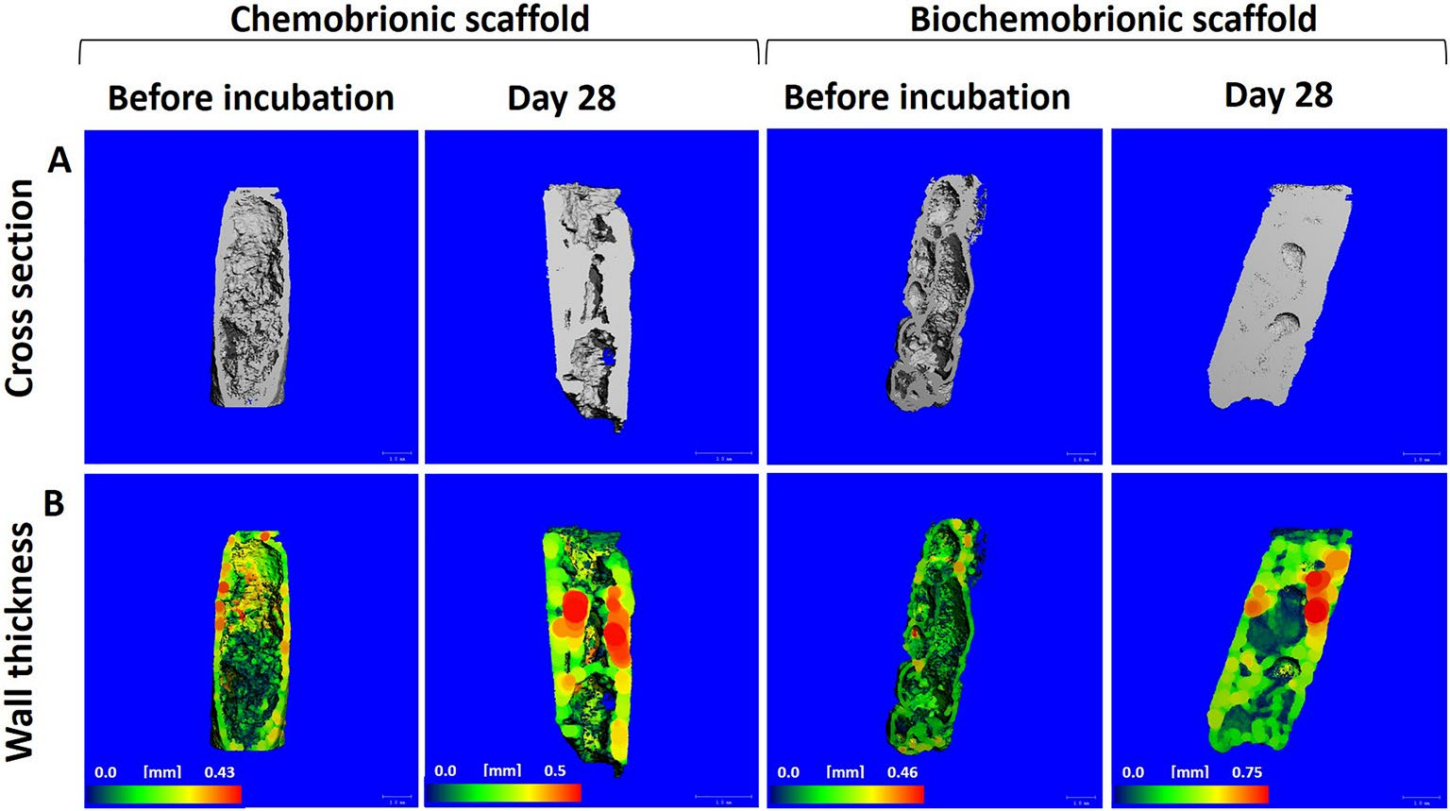
Micro-CT images of scaffolds in ODM before and after 28 days of the incubation.

Finally, the mineral deposition of CB and BCB scaffolds at day 28 was analyzed by micro-CT scans. According to scanning performed prior to in vitro experiments, the average pore size of CB and BCB was significantly different (p < 0.05), with values of 725 ± 104 µm and 430 ± 40 µm, respectively. There was no significant difference between (p > 0.05) the wall thickness of the CB (223 ± 14 µm) and BCB scaffolds (197 ± 23 µm). After analyses, scaffolds were sterilized, and MSCs were cultured onto these scaffolds in ODM. At 28 days, cross-sectional views and wall thickness results demonstrated that both scaffolds showed advanced mineralization starting from the surface through the center of the scaffolds (Fig. 8). Almost all the large voids found in the scaffolds were filled with newly formed minerals. This result was confirmed by the increasing wall thicknesses to 283 ± 12 µm and 273 ± 53 µm for CB and BCB scaffolds, respectively. A quantitative measurement for bone formation was also evaluated, considering the bone volume to total volume ratio (BV/TV). According to scanning, the BCB scaffold (BV/TV: 0.831 ± 0.009) showed significantly higher bone formation (p < 0.05) than that of the CB scaffold (BV/TV: 0.761 ± 0.041).

## Conclusion

In the present study, a new bioactive bone scaffold, called biochemobrionic, was developed by incorporating diatom BS into the CB structure. Additionally, the utilization of CB and BCB structures as bone scaffolds was extensively examined through in vitro cell culture experiments involving stem cells. The characterization results revealed that both structures exhibited favorable characteristics for their application in bone tissue regeneration. These included suitable surface textures, chemical composition, and degradation behavior. However, the incorporation of diatom BS into the CB structure resulted in an increase in silica content, porosity, biodegradation rate, and mechanical strength. In vitro culture studies showed that BCB scaffold provided higher differentiation efficiency, as evidenced by increased ALP activity, calcium deposition, relative gene expression, and mineralization. Also, the presence of diatom BS induced osteogenic differentiation even in the absence of specific osteogenic stimuli. Overall, BCB scaffold showed greater activity than CB scaffold due to the presence of diatom BS, which demonstrated the potential to induce the precipitation of calcium phosphate, stimulate osteoblast proliferation, and inhibit osteoclast formation. Incorporation of diatom BS into the CB structure is a promising strategy to enhance the osteogenic activity of scaffold, thus the developed BCB scaffold is considered a promising candidate for bone repairing. In the next stage, BCB scaffold can be implemented into animal models to validate the use for bone regeneration. Scaffolds for repairing bone defects should possess good mechanical properties, ideally matching or exceeding those of the surrounding bone to reconstruct hard load-bearing tissues. While the integration of BS into the CB structure improved its mechanical properties, it's noteworthy that the maximum compressive strength obtained was still lower than that of trabecular and cortical bones. Therefore, future studies may focus on the integration of nanoparticles, such as nanodiamonds, silver nanoparticles, or bioactive glass particles, to enhance the mechanical properties of BCB scaffold. Additionally, coating the scaffolds’ surface with different macromolecules (i.e. chitosan, gelatin, etc.) may be a promising approach to further strengthen it. From another perspective, the BCB scaffold may be a suitable candidate for structural support or as a bone void filler, particularly in applications where superior mechanical strength is not a primary requirement. Furthermore, BCB can also be used in different sectors that will benefit from its bioactive properties.

### Supplementary Information

Supplementary Information.

## Data Availability

Data will be made available on request.
