# Supplementary material for: Design of chemobrionic and biochemobrionic scaffolds for bone tissue engineering
Source: Sci Rep. 2024 Jun 14;14:13764. doi: 10.1038/s41598-024-63171-z (PMC11178857; doi:10.1038/s41598-024-63171-z)
Supplement: Supplementary file 1 — Supplementary Information. [file 41598_2024_63171_MOESM1_ESM.docx]

**Supplementary material**

**Table S1.** Primer sequences for osteogenic markers

| Primer name | Forward primer sequence 5'-3' | Reverse primer sequence 5'-3' |
| --- | --- | --- |
| OCN | AGGACCCTCTCTCTGCTCAC | AACGGTGGTGCCATAGATGC |
| OPN | CCGATGAATCTGATGAGTCCTT | TCCAGCTGACTTGACTCATGG |
| ON | GGAAGCTGCAGAAGAGATGG | TGCACACCTTTTCAAACTCG |
| ALP | TCCATGGTGGATTATGCTCA | TTCTGTTCCTGCTCGAGGTT |
| β-actin | GCAGATGTGGATCAGCAAGC | AAAGGGTGTAAAACGCAGCTC |


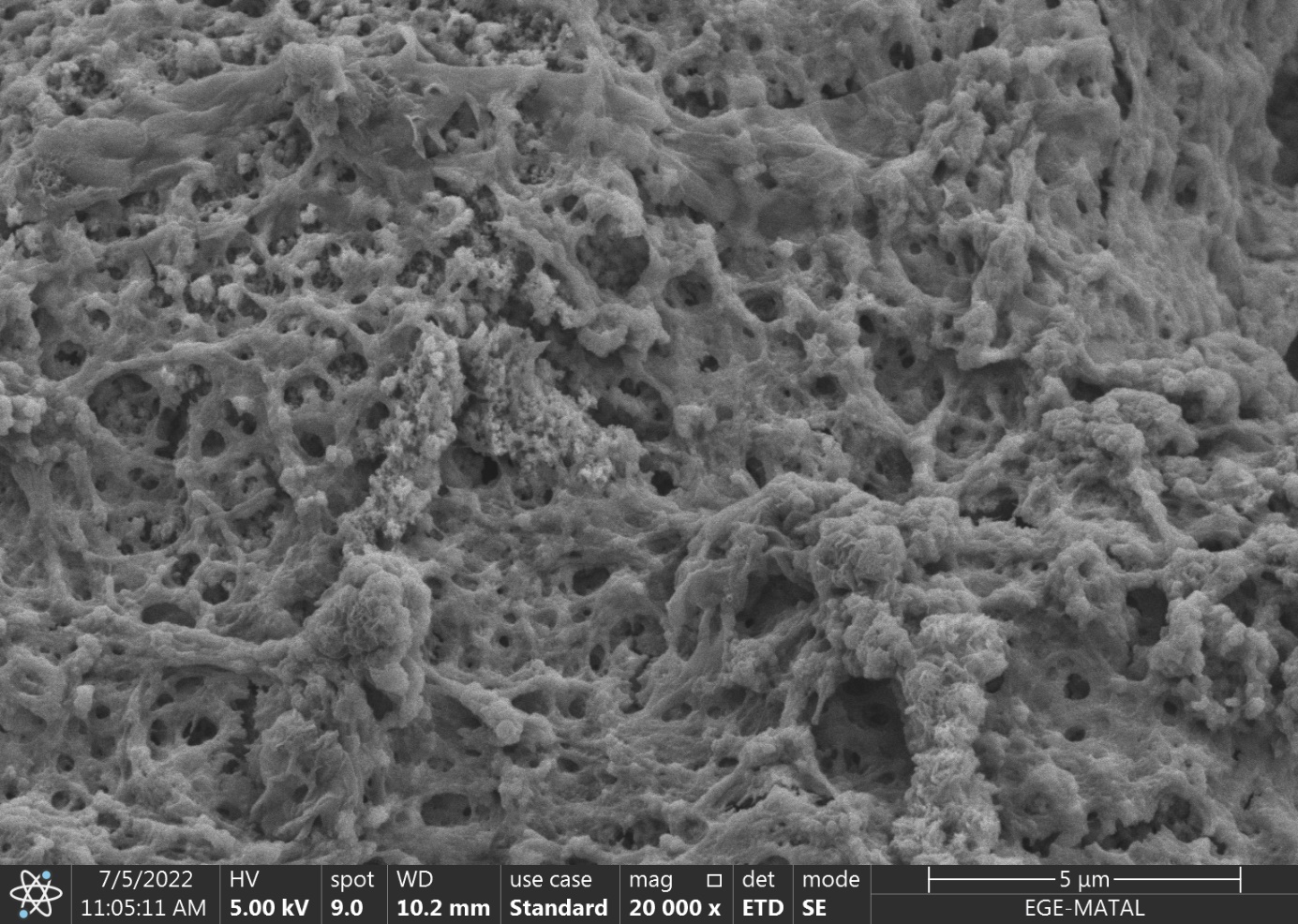


**Figure S1.** SEM images of MSCs on CB scaffold after 28 days of incubation in ODM


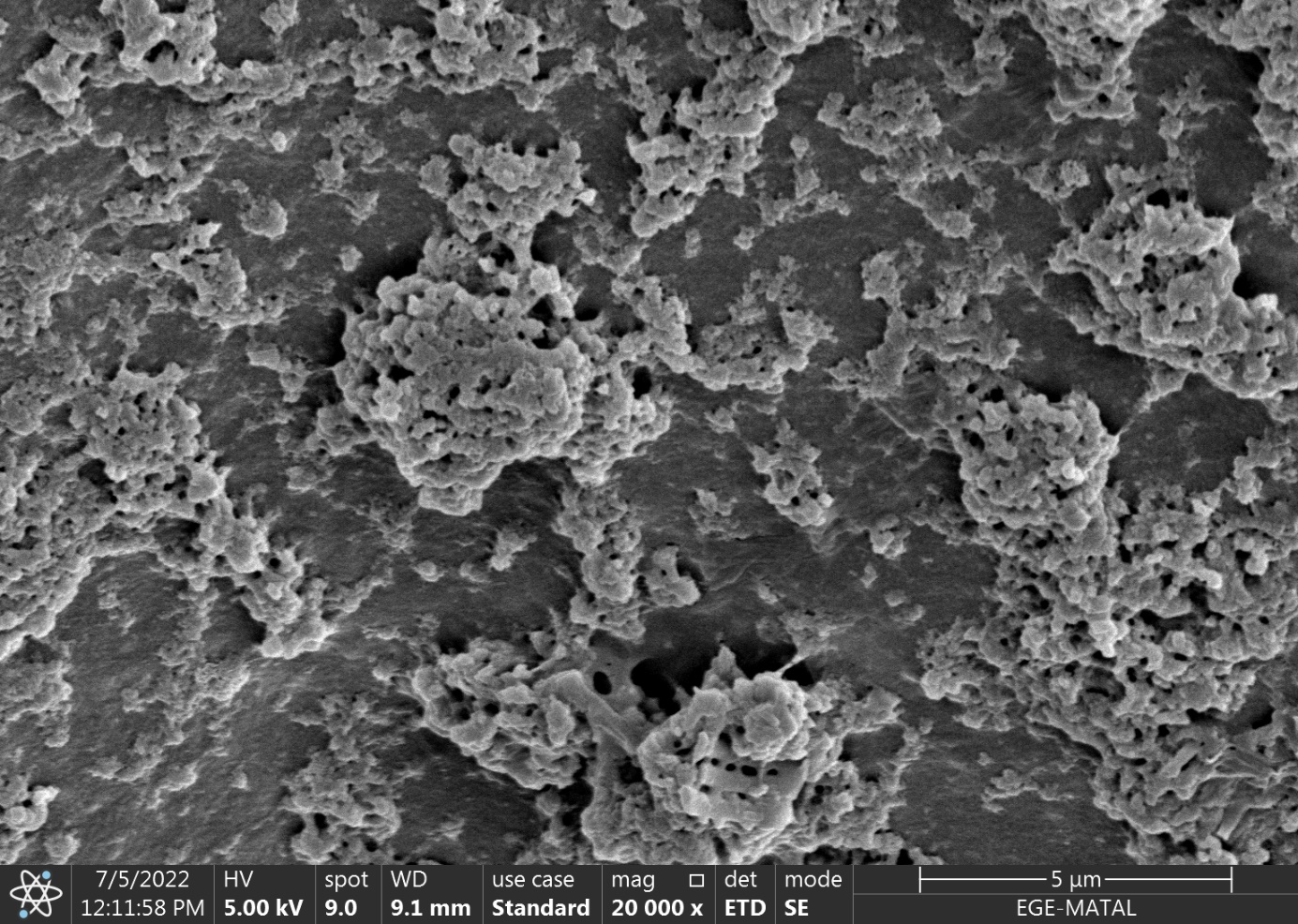


**Figure S2.** SEM images of MSCs on BCB scaffold after 28 days of incubation in ODM
